# Effectiveness of Personal Protective Measures to Prevent Lyme Disease

**DOI:** 10.3201/eid1402.070725

**Published:** 2008-02

**Authors:** Marietta Vázquez, Catherine Muehlenbein, Matthew Cartter, Edward B. Hayes, Starr Ertel, Eugene D. Shapiro

**Affiliations:** * Yale University School of Medicine, New Haven, Connecticut, USA; † State of Connecticut Department of Public Health, Hartford, Connecticut, USA; ‡ Centers for Disease Control and Prevention, Fort Collins, Colorado, USA

**Keywords:** Lyme disease, Lyme vaccine, personal protective measures, effectiveness, prevention, Borrelia burgdorferi, research

## Abstract

After the manufacture of Lyme vaccine was discontinued in 2002, strategies to prevent Lyme disease (LD) have focused on personal protective measures. Effectiveness of these measures has not been conclusively demonstrated. The aim of our case–control study was to assess the effectiveness of personal preventive measures in a highly disease-endemic area. Case-patients were persons with LD reported to Connecticut’s Department of Public Health and classified as having definite, possible, or unlikely LD. Age-matched controls without LD were identified. Study participants were interviewed to assess the practice of preventive measures and to obtain information on occupational and recreational risk factors. Use of protective clothing was 40% effective; routine use of tick repellents on skin or clothing was 20% effective. Checking one’s body for ticks and spraying property with acaricides were not effective. We concluded that use of protective clothing and of tick repellents (on skin or clothing) are effective in preventing LD.

## Abstract 2

Use of protective clothing and tick repellents on the skin or clothing while outdoors is 40% and 20% effective, respectively.

Lyme disease (LD) is the most commonly reported tick-borne illness in the United States (*1*,*2*). A vaccine against the disease was licensed in 1998 for use in persons 15–70 years of age (*3*). However, because of poor sales, it was withdrawn from the market in 2002. Recommendations for preventing LD currently focus on personal protective measures and interventions to reduce abundance of vector ticks (*4*). Strategies include avoidance of tick-infested areas, use of protective clothing (i.e., wearing long-sleeved shirts and long pants, which decrease the area of exposed skin), routine checks of one’s body for ticks, and the use of tick repellents on either the skin or clothing. Other strategies have targeted the environment and vertebrate hosts of deer ticks; however, such approaches are often impractical, and their effects on the incidence of LD are unknown. Finally, use of antimicrobial prophylaxis in selected persons who have been bitten by a deer tick may be effective (*5*).

Although personal protective measures are frequently recommended by medical providers, public health officials, and the lay press in areas where the disease is endemic, few data about their effectiveness exist. In 1992, Herrington et al. (*6*) found that self-perceived risk of acquiring LD and knowledge about the disease correlated with the use of personal protective measures; however, even in highly LD-endemic areas such as Connecticut, adherence to these personal protective measures varied greatly—only 79% of respondents routinely used tick repellents and 93% reported inspecting themselves for and removing ticks after being outdoors. In 2001, Phillips et al. found that 80% of the surveyed residents of Nantucket reported checking their bodies for ticks after potential tick exposures, 53% used protective clothing (such as wearing long pants and long-sleeved shirts), 34% reported routinely avoiding tick infested areas, and 11% reported routine use of tick repellents (*7*). These researchers did not find a difference in reported frequency of LD among those who did and those who did not report preventive behavior. Similarly, Orloski et al. did not find any statistically significant differences in use of protective measures between persons with LD and age-matched controls (*8*).

Although certain occupations, such as working outdoors in forestry or landscaping, have been suggested to increase the risk for LD, few studies document increased risk among such workers. Smith et al. evaluated outdoor workers by using questionnaires and serologic tests for antibody to *Borrelia burgdorferi* and found that workers with a history of outdoor employment were twice as likely to be seropositive as those without such a history; however, this difference was not statistically significant (*9*). A study conducted in California by Lane et al. found that outdoor occupations such as woodcutting were associated with an increased risk for LD (*10*).

We conducted a matched case–control study of persons 15–70 years of age residing in Connecticut. The purpose was to assess the effectiveness of Lyme vaccine and personal protective measures against LD. The study ceased soon after Lyme vaccine was withdrawn from the market.

## Methods

### Study Population

We conducted a matched case–control study from July 2000 to February 2003. Study participants were identified through an active surveillance system for LD initiated in 1991. Physicians in private practice and other health maintenance organizations throughout the state participated in the surveillance system. Staff of the State of Connecticut Department of Public Health (CT-DPH) contacted healthcare providers regularly to inquire about newly diagnosed cases of LD. Supplemental follow-up reporting forms were also sent to physicians when additional clinical information was needed. The study was approved by the Human Investigations Committee at the Yale University School of Medicine and the Institutional Review Boards of the CT-DPH and the Centers for Disease Control and Prevention (CDC).

Potential case-patients were persons 15–70 years of age (because Lyme vaccine was approved for persons in this age range) reported by their healthcare provider to CT-DPH as having LD in the period from January 2000 to February 2003. Study participants were enrolled prospectively (i.e., as each case was identified, case-patients and controls were recruited and enrolled). Letters of invitation were sent to potential case-patients after the reporting physician gave permission to contact the patient.

Controls were persons 15–70 years of age without LD who were matched to the case-patients by age (±5 years from case-patient’s birth date) and who had a telephone. Controls were identified by using sequential-digit dialing, a technique that uses sequential digits from the end of the telephone number of the case-patient to contact potential controls (*11*). This technique also ensured that the case-patient and control resided in the same general geographic area. To maximize the likelihood of contacting control study participants, telephone calls were made on nonconsecutive days, including at least 1 weekend day (during daytime and evening hours). For each case-patient, we enrolled up to 2 matched controls. Controls were excluded if they had had LD 30 days before the diagnosis of LD in the matched case-patient (as stated either by them during the interview or as documented in their medical record), but neither case-patients nor controls were excluded if they had had LD in the more distant past, since a previous history of the disease was not a contraindication for the vaccine and LD can be acquired more than once (*12*).

### Data Collection

Trained personnel obtained informed consent and interviewed both case-patients and controls by telephone with a standardized survey. For study participants <18 years of age, a parent or guardian was interviewed. Case-patient study participants were interviewed within the year of their Lyme diagnosis. Questions concerning demographics, occupational (forestry or landscaping) and recreational risk factors (camping or other outdoor activities), personal protective measures (specifically the use of tick repellents on the skin or clothing while outdoors; spraying one’s property with acaricides; use of protective clothing such as long pants, long-sleeved shirts, and light-colored clothing; and checking one’s body for ticks after being outdoors) to prevent LD were asked of all study participants; for case-patients, questions about clinical signs and symptoms were asked. The questions were phrased to discuss behavior of the case-patient before the diagnosis of LD; for control study participants, we asked about behavior before the date in which their matched case was diagnosed. For example, for a case-patient who received a diagnosis in July 2002, we asked him or her to respond to the questions on behavior before the LD diagnosis in July 2002: “Do you check your body routinely for ticks after outdoor exposure?” For the case-patient’s matched control, we asked the same question, using the last 12 months before the diagnosis of the case as the reference period. Questions are shown in Table 1.

**Table 1:** Questions asked in the survey regarding risk factors and personal protective measures

Do you (your child) live in close proximity to grassy fields or heavily wooded areas?
Do you (your child) have an occupational exposure that puts you at risk for tick bites (such as working in landscaping, forestry, farming, or wild-life parks management)?
Do you (your child) engage in outdoor activities that put you at higher risk for tick bites (such as hiking, camping, gardening, hunting)?
Do you (your child) wear clothing to protect against ticks while outdoors, e.g., long pants, long-sleeved shirts, or light-colored clothing?
Do you (your child) routinely use tick repellents on the skin and/or clothing while outdoors?
Do you routinely spray acaricides against ticks on your property?
Do you (your child) routinely check for ticks on the body after being outdoors?
Do you have any pets at home?

The medical records of case-patients and matched controls were reviewed. Information was recorded about receipt of Lyme vaccine (a case-patient was considered vaccinated if at least 1 dose of Lyme vaccine was received >30 days from the date of the LD diagnosis; for controls, we used the dates of the matched case-patient), previous medical history and, for case-patients, clinical and laboratory data at the time of diagnosis. Case-patients and controls were excluded from the analysis if their medical records could not be obtained.

### Classification of LD

Case-patients were classified into 3 categories by 2 investigators, who were blinded to the case-patient’s name, age, source of medical care, vaccination status, and use of personal protective measures; disagreements were resolved by discussion. The category of definite LD included study participants who met the surveillance case definition, i.e., erythema migrans (EM) measuring at least 5 cm in diameter documented by a physician, objective signs of early disseminated disease with a positive test result for antibodies against *B. burgdorferi* (if EM was not present), or objective signs of late disease with a positive test for antibodies against *B. burgdorferi* (the patient must have had antibodies measured by using the 2-tiered system recommended by CDC). The category of possible LD included those study participants who met most, but not all, of the criteria in the surveillance case definition, such as a case with EM that was either <5 cm in diameter or a size that was not documented. The third category included those study participants who were unlikely to have LD, i.e., they had only nonspecific symptoms, such as fatigue, and case-patients with objective findings but with negative serologic test results for LD. In addition, we classified all reported cases of LD according to the clinical stage of LD at the time of the diagnosis: early localized disease (single EM), early disseminated disease (multiple EM, early neurologic disease or cardiac disease), or late LD (arthritis or encephalomyelitis).

### Statistical Analysis

Matched odds ratios (ORs), associated 95% confidence intervals (CIs), and their statistical significance were calculated by using SAS software (SAS Institute, Cary, NC, USA). Conditional logistic regression (EGRET Windows; CYTEL Corp., Cambridge, MA, USA) was used to adjust the matched ORs for the effects of potential confounding factors, including sex, use of other personal protective measures, receipt of Lyme vaccine, and race. Effectiveness was calculated as 1 = the matched OR; p values <0.05 were considered significant.

## Results

We identified 1,436 age-eligible potential case-patients from January 2000 to February 2003. Thirty-two reported case-patients (2%) were not contacted because the reporting physician refused to permit it. Of the remaining 1,404 case-patients, we were unable to contact 340 (24%). Of the 1,064 who were contacted, 195 (18%) refused to participate (most refused to give consent to review their medical records). We enrolled and interviewed the remaining 869 potential case-patients (82%). Of those enrolled in the study, matched controls were not identified for 78 (9%), and data were incomplete for 82 (9%) (i.e., medical records could not be found). Data presented were part of a larger study of the effectiveness of Lyme vaccine. The study ended when the Lyme vaccine was withdrawn from the market and controls had not yet been obtained for all study participants.

Characteristics of the 709 case-patients in the study who had at least 1 matched control and the 1,128 matched controls are shown in Table 2. The age at the time of infection ranged from 15 to 70 years (median 48 years). Of the 709 cases (419 cases had 2 controls and 290 had 1 control), 467 (66%) were classified as definite cases of LD, 105 (15%) as possible cases, and 137 (19%) as unlikely to be LD. We found controls were more likely to be female; this difference could be explained by our method of identification and enrollment of controls (by telephone), if women were more likely to be at home at the time of our call. Similar proportions of case-patients and matched controls had received Lyme vaccine.

**Table 2:** Characteristics of Lyme disease study participants, Connecticut, July 2000 through February 2003

Characteristics	Case-patients (N = 709), no. (%)	Controls (N = 1,128), no. (%)	p value
Age, y			
Median	48	49	0.71
Mean	46	47	
Range	15–70	15–70	
Sex			
Female	376 (53)	715 (63)	<0.001
Male	333 (47)	413 (37)	
Race			
Caucasian	689 (97)	1094 (97)	0.66
African American	3	8	
Hispanic	4	6	
Other	13	14	
Underlying medical problems other than Lyme disease (e.g., diabetes, asthma)	298 (42)	508 (45)	0.19
Had Lyme disease*	110 (17)	143 (14)	0.095
Received Lyme vaccine†	44 (6)	73 (6)	0.82

*Having Lyme disease was defined as the following: for case-patients, having Lyme disease at a time other than the episode for which a case-patient was enrolled in the study; for controls, having Lyme disease at a time other than the focal time of disease for the case-patient. †A study participant was considered vaccinated if he or she received at least 1 dose of Lyme vaccine.

Tables 3 and 4 show risks factors for and use of personal protective measures against LD for case-patients, stratified by certainty of the diagnosis, and their matched controls. Adjustment for potential confounding variables such as gender, race, receipt of Lyme vaccine, and use of other personal protective measures was made with conditional logistic regression. Patients classified as having either definite or possible cases of LD (N = 572) were not significantly more likely to report risk factors for LD than their matched controls (N = 898). Definite and possible case-patients were less likely than controls to report using protective clothing outdoors (46% vs. 58%; adjusted OR 0.6, 95% CI 0.5–0.8; effectiveness 40%, p<0.0001) and to use tick repellents on their skin or clothing (29% vs. 34%, adjusted OR 0.8, 95% CI 0.6–1.0; effectiveness 20%, p = 0.05). Spraying acaricides routinely on one’s property did not differ significantly for case-patients and matched controls. Estimates did not change significantly when only those cases classified as definite LD were analyzed (Table 3).

**Table 3:** Personal protective measures and risk factors for Lyme disease, Connecticut, July 2000 through February 2003

Personal protective measures	Case-patients, no. (%)	Matched controls, no. (%)	Odds ratio* (95% CI)†	p value
Use of protective clothing while outdoors				
Definite	215 (46) N = 467	427 (59) N = 724	0.6 (0.5–0.7)	<0.0001
Definite and possible	265 (46) N = 572	524 (58) N = 898	0.6 (0.5–0.8)	<0.0001
Unlikely	72 (53) N = 137	121 (53) N = 230	0.9 (0.6- 1.3)	0.55
Use of tick repellents on skin or clothing while outdoors				
Definite	138 (30) N = 467	252 (35) N = 724	0.8 (0.6–0.9)	0.04
Definite and possible	168 (29) N = 570	303 (34) N = 890	0.8 (0.6–0.99)	0.0499
Unlikely	37 (27) N = 136	64 (28) N = 228	0.9 (0.6–1.5)	0.83
Spraying property with tick acaricides				
Definite	16 (7) N = 237	52 (11) N = 467	0.6 (0.3–1.1)	0.09
Definite and possible	19 (7) N = 285	62 (11) N = 557	0.6 (0.3–1.0)	0.06
Unlikely	3 (8) N = 36	16 (12) N = 133	0.7 (0.2–3.0)	0.61
Checking the body for ticks after exposure				
Definite	360 (77) N = 467	560 (77) N = 724	1.1 (0.8–1.4)	0.64
Definite and possible	443 (77) N = 572	703 (78) N = 898	1.0 (0.8–1.4)	0.81
Unlikely	107 (78) N = 137	181 (79) N = 230	0.9 (0.5–1.5)	0.61

*All estimates were adjusted for possible confounders (sex, race, receipt of Lyme vaccine, and use of other personal protective measures) with conditional logistic regression. †CI, confidence interval.

**Table 4:** Risk factors for Lyme disease, Connecticut, July 2000 through February 2003

Risk factors	Case-patients, no. (%)	Matched controls, no. (%)	Odds ratio* (95% CI†)	p value
Having an occupational exposure				
Definite	68 (15) N = 462	80 (11) N = 699	1.4 (0.9–2.0)	0.074
Definite and possible	87 (15) N = 566	109 (13) N = 866	1.2 (0.9–1.6)	0.32
Unlikely	38 (28) N = 136	27 (12) N = 222	3.1 (1.6–5.9)	0.0007
Engaging frequently in outdoor activities				
Definite	400 (86) N = 467	598 (83) N = 724	1.2 (0.9–1.7)	0.26
Definite and possible	489 (85) N = 572	745 (83) N = 898	1.2 (0.9–1.6)	0.34
Unlikely	117 (85) N = 137	197 (86) N = 230	0.9 (0.5–1.7)	0.78
Living close to grassy or heavily wooded area				
Definite	444 (95) N = 467	676 (93) N = 724	1.4 (0.8–2.3)	0.23
Definite and possible	546 (95) N = 572	841 (94) N = 898	1.4 (0.9–2.3)	0.18
Unlikely	131 (96) N = 137	218 (95) N = 230	0.9 (0.3–2.6)	0.89
Having pets at home				
Definite	283 (73) N = 386	488 (72) N = 681	1.2 (0.9–1.7)	0.17
Definite and possible	355 (75) N = 472	599 (71) N = 838	1.2 (0.9–1.6)	0.17
Unlikely	81 (77) N = 105	151 (72) N = 210	1.4 (0.8–2.6)	0.27

*All estimates were adjusted for possible confounders (sex, race, receipt of Lyme vaccine, and use of other personal protective measures) with conditional logistic regression. †CI, confidence interval.

Recreational outdoor activities, such as hiking and camping (85% vs. 83%, p = 0.34), living near heavily wooded or grassy areas (95% vs. 94%, p = 0.18), and having pets at home (75% vs. 12%, p = 0.17), were not statistically significantly associated with LD in any of the groups. Only among groups in which the case-patient was classified as unlikely to have LD were patients significantly more likely than matched controls to have an occupational risk factor for LD such as forestry or landscaping (28% vs. 12%, adjusted OR 3.1, 95% CI 1.6–5.9, p = 0.0007). None of the personal protective measures was effective in groups classified as unlikely to have LD.

We also analyzed the data excluding all controls with a previous history of LD, and the results were virtually unchanged except that having an occupational exposure was a risk factor. When we excluded controls who had previously had LD from the analysis, case-patients were more likely to have an occupational exposure than were matched controls; this was true for definite cases of LD (60 [15%] of cases vs. 51 [9%] of controls; OR 1.5, 95% CI 0.9–2.4, p = 0.05), for definite cases and possible cases (78 [16%] of cases vs. 69 [10%] of controls; OR 1.5, 95% CI 1.03–2.2, p = 0.03), and for unlikely cases (34 [28%] of cases vs. 16 [9%] of controls; OR 3.6, 95% CI 1.8–7.7, p = 0.0006).

## Discussion

This is the first study, to our knowledge, to demonstrate that any personal protective measure against LD is effective. Use of protective clothing while outdoors was 40% effective in strata with case-patients classified as having definite or possible LD. Of note, this strategy was not significantly effective in strata with case-patients classified as unlikely to have LD (not only were the differences between patients and controls in this group not statistically significant, but the magnitude of the effect was much diminished), which supports the validity of the results (*13*). The use of tick repellents on the skin or clothing while outdoors was also effective (effectiveness of 20%) for preventing LD.

By contrast, inspecting one’s body for and removing ticks was not found to be an effective strategy to prevent LD nor was using acaricides on one’s property. A potential limitation of the study is that use of the protective measures was based on self-report, so these practices could neither be confirmed nor quantified. Undoubtedly, there is variability in the practice of these personal protective measures that was not ascertained by the study. For example, some persons may only perform cursory tick checks, while others may engage in a more careful examination. Similarly, there may be variability in the application of tick repellents—in the amount and frequency of application and whether the repellents were applied to clothing, to exposed skin, or to both, as well as variations in repellents themselves. (Active ingredients such as DEET [N,N-diethyl-3-methyltoluamide], permethrin, or natural or herbal repellents and their concentrations may vary.) Whether rigorous practice of these protective measures might be protective is unknown. Furthermore, we did not assess for ecologic differences that may have played a role in the possibility for household acaricides to have been effective or for risk factors for the disease, such as apartment versus single family dwelling. The effectiveness of the use of acaricides may also possibly rely on routine use not only by the study participant but also by close neighbors.

We used sequential-digit technique, which ensures that controls are generated from the same general area. We did not analyze differences in locations within telephone exchanges; however, LD is considered to be endemic in the entire state of Connecticut, and no major differences would be expected within areas of a telephone exchange. Another potential limitation of our study is recall bias—study participants are more likely to remember things related to LD if they have had the disease itself.

The finding that occupational exposure did not appear to be a risk factor for cases classified as definite or possible LD suggests that most of the case-patients with true LD reported in Connecticut acquired their LD from periresidential or recreational exposure. However, when we excluded controls with a history of LD in the past, occupational exposure was a risk factor for the disease. The finding that case-patients classified as unlikely to have LD were more likely to have had occupational exposures than the corresponding controls is somewhat perplexing. Possibly persons who are in occupations that have been associated with LD and who are aware of their potential risk for the disease are more likely to seek medical attention for suspected LD symptoms, and their physicians may be more likely to report them as LD cases because of the perceived occupational risk. Such a diagnostic bias would tend to artificially elevate the prevalence of occupational exposure among these persons suspected to have LD who in fact probably do not have LD. It is also possible that occupational exposure is a true risk factor for the other conditions that are causing symptoms in these persons who are unlikely to have LD.

Public health strategies are important in the control of LD, an emerging infection with continually increasing incidence (*1*); nonetheless, the implementation and assessment of these strategies have proven to be challenging. Public health officials must take into account not only the effectiveness of the public health strategy, but also the level of engagement of those who are supposed to follow the recommendations (*14*). Although this study indicates that use of protective clothing and the use of tick repellents on the skin or clothing while outdoors are effective, clearly not all persons at risk follow these recommendations. Our results provide data to support recommendations that have been in place for many years. By no means should our results be taken as recommendations to cease personal protective measures that have been recommended by public health officials but that we found not to be effective. Although our study included adolescents, most study participants were adults; therefore, whether these findings apply to children, a group at high risk of acquiring LD, is not known.

Additional educational efforts about these practices, targeted at persons living in LD-endemic areas, may be beneficial. The use of protective clothing may be important for preventing not only LD but also other tick-borne infections. Nevertheless, these strategies, even used optimally, are likely to prevent only a portion of cases of LD. Other strategies, such as improved vaccines and measures to reduce tick abundance in areas where human exposure to ticks is high, should continue to be pursued.
